# Thalamic ventral-Oralis complex/rostral zona incerta deep brain stimulation for midline tremor

**DOI:** 10.1007/s00415-024-12619-3

**Published:** 2024-08-10

**Authors:** Alba Scerrati, Andrea Gozzi, Michele Alessandro Cavallo, Giorgio Mantovani, Pietro Antenucci, Chiara Angelini, Jay Guido Capone, Pasquale De Bonis, Francesca Morgante, Vittorio Rispoli, Mariachiara Sensi

**Affiliations:** 1 Department of Translational Medicine, University of Ferrara, Ferrara, Italy; 2 Neurosurgery Department, S. Anna University Hospital of Ferrara, Ferrara, Italy; 3 Neurology Department, S. Anna University Hospital of Ferrara, Via Aldo Moro 8, 44124 Ferrara, Italy; 4 Neurosciences and Cell Biology Institute, Neuromodulation and Motor Control Section, St George’s University of London, London, UK; 5 Neurology, Neuroscience Head Neck Department, University of Modena and Reggio Emilia, Modena, Italy; 6 Department of Neuroscience and Rehabilitation, University of Ferrara, Ferrara, Italy via Aldo Moro 8, 44124

**Keywords:** Midline tremor, Dystonia, DBS, Ventral-Oralis complex, Zona Incerta, Thalamus

## Abstract

**Background:**

Midline Tremor is defined as an isolated or combined tremor that affects the neck, trunk, jaw, tongue, and/or voice and could be part of Essential Tremor (ET), or dystonic tremor. The clinical efficacy of deep brain stimulation for Midline Tremor has been rarely reported. The Ventral Intermediate Nucleus and Globus Pallidus Internus are the preferred targets, but with variable outcomes. Thalamic Ventral-Oralis (VO) complex and Zona Incerta (ZI) are emerging targets for tremor control in various etiologies.

**Objective:**

To report on neuroradiological, neurophysiological targeting and long-term efficacy of thalamic Ventral-Oralis complex and Zona Incerta deep brain stimulation in Midline Tremor.

**Methods:**

Three patients (two males and one female) with Midline Tremor in dystonic syndromes were recruited for this open-label study. Clinical, surgical, neurophysiological intraoperative testing and long-term follow-up data are reported.

**Results:**

Intraoperative testing and reconstruction of volume of tissue activated confirmed the position of the electrodes in the area stimulated between the thalamic Ventral-Oralis complex and Zona Incerta in all patients.

All three patients showed optimal control of both tremor and dystonic features at short-term (6 months) and long-term follow-up (up to 6 years). No adverse events occurred.

**Conclusion:**

In the syndromes of Midline Tremor of various origins, the best target for DBS might be difficult to identify. Our results showed that thalamic Ventral-Oralis complex/Zona Incerta may be a viable and safe option even in specific forms of tremor with axial distribution.

**Supplementary Information:**

The online version contains supplementary material available at 10.1007/s00415-024-12619-3.

## Introduction

Midline Tremor refers to an isolated or combined tremor that affects the neck, trunk, jaw, tongue, and/or voice. Midline Tremor may arise in the context of Essential Tremor (ET) or manifest in the head and trunk combined with dystonia [1–4].

Deep brain stimulation (DBS) of the Ventral Intermediate Nucleus (VIM) is considered the treatment of choice for ET, and some beneficial effects have also been reported in selected cases of midline ET [5]. The combination of dystonia and tremor (defined as dystonic tremor or tremor associated with dystonia, depending whether dystonia and tremor overlap on the same body district) is a therapeutic challenge. Both Globus Pallidus Internus (GPi) and VIM have been reported as effective targets for dystonic tremor (DT) [6, 7]. However, GPi-DBS, which is a consolidated and effective procedure for dystonia, does not always relieve tremor [8]. Conversely, VIM-DBS is highly effective for action tremors, but there is poor evidence about the effect on dystonia. Rarely, VIM-DBS might worsen dystonia [9] and a recent case series showed two cases with midline DT failing VIM-DBS and being rescued with GPi-DBS [8]. Finally, double targeting of GPi and VIM has been rarely reported in selected patients and has shown effectiveness [10]. These studies, including a review of the literature [8], have highlighted the lack of consensus on which are the best DBS targets for the combination of dystonia and tremor.

Recently, DBS of the thalamic Ventral-Oralis (VO) complex, which includes both Ventralis Oralis anterior (VOa) and Ventralis Oralis posterior (VOp) thalamic nuclei (VOa–VOp complex), has been reported to be effective for dystonic tremor [11]. Considering its pallido-thalamic and cerebello-thalamic afferents, VO complex is a potential target in different refractory tremor syndromes [12].

Limited data exist regarding the efficacy and safety on Zona Incerta (ZI) DBS for tremor syndromes. The rationale for using this target is still debated for many reasons. First, nomenclature confusion still exists regarding the anatomical differentiation between rostral (r-ZI) or caudal (c-ZI) Zona Incerta, with the latter often referred to as part of the Posterior Subthalamic Area (PSA). Second, different groups used different surgical techniques to target ZI [13]. For this reason, targeting this area lacks standardization. Finally, DBS programming is often not comparable among different groups, making it unclear whether these two zones are equivalent in efficacy and safety for DBS in tremor syndromes. Moreover, there is a lack of data in the literature on double targeting of ZI and VOa–VOp complex in different forms of non-parkinsonian tremor [14].

Here we report the long-term outcomes of three patients with Midline Tremor as the predominant symptom, treated with DBS targeting with the same lead the VOa–VOp complex and ZI. In two of these patients, the Midline Tremor was part of isolated dystonia, while in one, dystonia was only a soft sign and was associated with ataxic gait [15]. VOa–VOp complex was preferred over GPi because tremor was the most prominent and disabling symptom. VOa–VOp was also chosen instead of VIM, as this target has been reported to be effective for dystonia in subjects with DT [11]. We also chose to target ZI, considering the recent reports about its efficacy in various tremor syndromes [13, 16–23].

## Methods

Inclusion criteria for continuous VO complex/ZI-DBS were: age 18 to 85 years, tremor syndromes refractory to pharmacotherapy, no other psychiatric/neurological disease or cognitive deficits, and patients’ informed consent.

Demographic characteristics, clinical, neurophysiological intraoperative testing, and neuroradiological data were collected.

### Pre- and post-operative neurological evaluation

All patients underwent a thorough multidisciplinary evaluation, including assessments by movement disorder neurologists, neuropsychologists, and neurosurgeons, to determine surgical candidacy. Patients were evaluated before surgery and during follow-up, and tremor and dystonia were rated using the Fahn–Tolosa–Marsden Rating Scale (FTMTRS) and the Burke–Fahn–Marsden Dystonia Rating Scale Motor Score (BFMDRS-M).

Programming of the pulse generator was performed at the first post-operative visit, approximately 2 weeks after leads placement. The selection of DBS active contacts was based on the data of neurophysiologic intraoperative testing (microelectrode recording and intraoperative microstimulation), as well as general guidelines and algorithms on programming found in literature on tremor and dystonia [24–26]. A monopolar review was conducted, and tremor improvement as well as side effects were recorded for each individual contact. The follow-up visits for programming were scheduled between 1 and 3 months based on symptom severity and degree of improvement.

### Surgical procedure

**Fig. 1:**
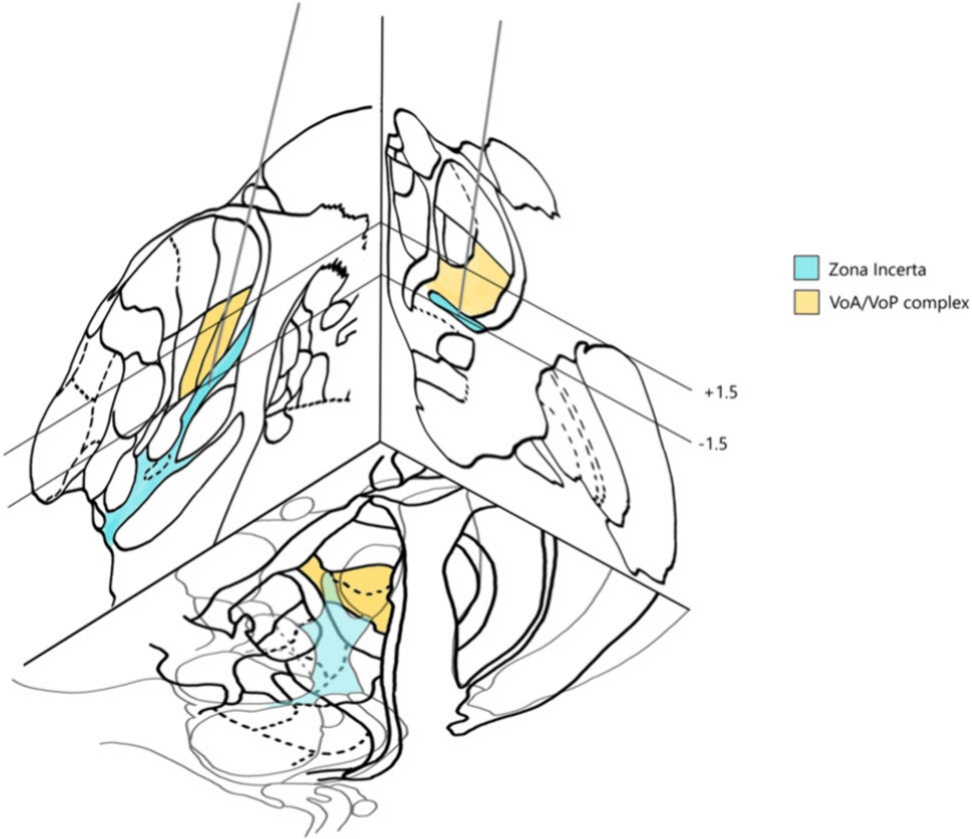
Schematic drawing of the selected target based on Schaltenbrand–Wahren atlas

All procedures were performed by the same neurosurgeon (M.A.C.) with the patient awake. A Leksell stereotactic frame was applied under local anesthesia, and patients underwent pre-operative Magnetic Resonance Imaging (MRI), acquiring contrast-enhanced T1 and T2 sequences. Considering that this target selection can be difficult due to the lack of visualization of the VOa–VOp complex and ZI on MRI, various indirect targeting strategies were employed. The stereotactic atlases (Schaltenbrand–Wahren atlas) [27] and proportional geometric schemes were used based upon measurements taken from adjacent structures (Fig. 1).

### Intraoperative recording and stimulation

Extracellular multi-unit recordings were performed in awake patients using a FHC microelectrode model 22,670 (FHC, Bowdoinham, ME, USA) mounted on a sliding canula. Microelectrode recording (MER) was conducted with a manual microdrive starting 6–8 mm before reaching the MRI-calculated target and extending 1.5–2.5 mm below the target in 0.5 mm increments along parasagittal simultaneous trajectories (central, anterior, and posterior) traversing VOp, VOa, and ZI. Thalamic neurons were tested for responses to voluntary movement, passive joint movements and cutaneous stimulation of the body surface to confirm the target within the anteroventral thalamic nuclei and eventually VIM pattern in the posterior track. The patterns encountered while passing through the VO complex, as described in the literature [28, 29], include two typical cell subpopulations: sporadic cells with a high firing rate (16.5 Hz) and low (5.5 Hz), or high firing rate (15–50 Hz) bursting type cells that could respond to voluntary movement. ZI is characterized by relative electrical silence interrupted by occasional spikes of isolated cells or cells discharging in a regular pattern at high amplitude at 25–45 Hz. Intraoperative microstimulation (IOM) with trains of 10-s electrical stimuli (frequency: 130 Hz; 60 μs square pulse, 5.0 mA) was performed to test for stimulus-related beneficial clinical effects (tremor reduction) and side effects (corticospinal/corticobulbar tract motor contractions and/or paresthesias). The final trajectory was chosen according to the track with the best recording pattern with the test electrode (multi-unit activity over a length of a minimum of 2.0 mm) and the absence of side effects and/or tremor reduction at semi-microstimulation. Intraoperative macrostimulation was also performed with the final DBS leads to confirm the chosen final tracks, using 0.5 mA increments up to 4.0 mA in a bipolar fashion, evaluating clinical effects and side effects at each of the four contacts (paresthesias, motor contractions, ataxia, dysarthria). The tip of the stimulating electrode was inserted 1.7 to 2.0 mm beyond the selected target (Table 1).

**Table 1:** Target coordinates referred to the AC-PC plane and midcommissural point

	*X* (mm)	*Y* (mm)	*Z* (mm)	Electrode tip (mm)
Case 1	10	− 3	0	+ 1.7
Case 2	12	− 1	− 4	+ 2.0
Case 3	12	0	+ 1	+ 2.0

Post-operative MRI was performed on the same day to confirm the correct position of the electrodes. On the first post-operative day, the infraclavicular internal pulse generator (IPG) was connected to intracranial leads via subcutaneous extension wires under general anesthesia.

### Imaging data analysis

**Fig. 2:**
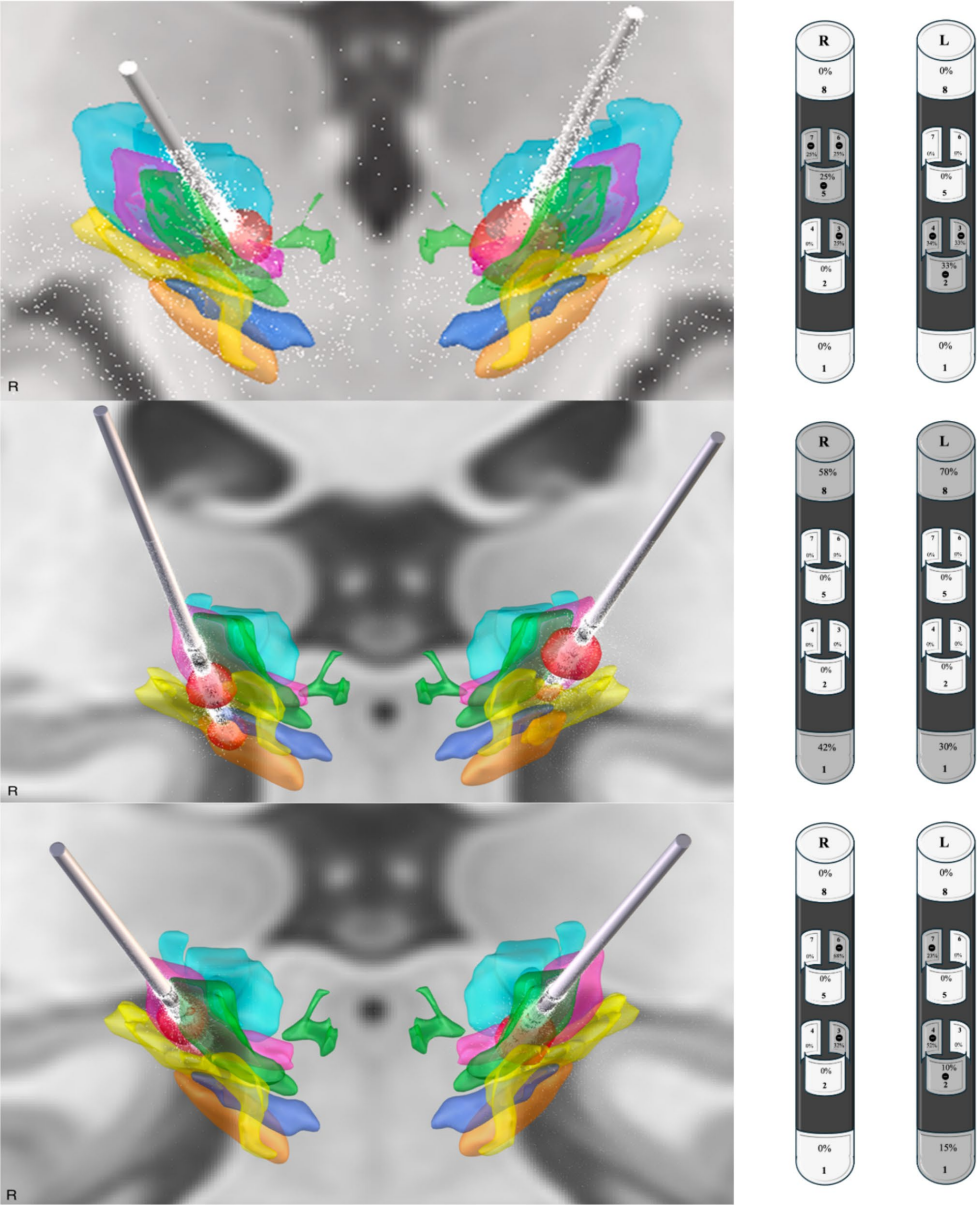
Leads and volumes of tissue activated (VTAs) in 3D rendering in case 1 (superior row), case 2 (middle row), and case 3 (inferior row). VTA was estimated according to the most effective and stable stimulation program. VTA: red. VOa: green. VOp: purple. VIM: light blue. Fields of Forel: blue. Zona Incerta: yellow. Subthalamic Nucleus: orange. On the right, a schematic representation of the electrodes with their respective stimulation parameters (activated contacts shown in gray)

Imaging data analysis was performed using custom software within Matlab^®^. Pre-operative high-resolution T1- and T2-weighted MRI images were co-registered and normalized to post-operative Computed Tomography (CT) scans with the Matlab Lead DBS Toolbox^®^ and SPM12. Correction for brain shift was applied to each co-registered and normalized image. The images were reconstructed using the Distal atlas [30], selecting VIM, VOa, VOp, ZI, fields of Forel and Subthalamic Nucleus (STN) as items of interest. The volumes of tissue activated (VTA) were visualized by setting parameters in the lead DBS software to stabilize stimulation (at 6-month post-implantation) (Fig. 2).

## Results

We included three subjects (one female, two males). Supplementary figure shows sequential axial sections of post-operative high-resolution T2-weighted MRI images for each patient reported (MRI images in supplementary material, Online Resource 1).

### Case 1

This 69-year-old woman underwent bilateral VOa-VOp/ZI DBS implant (Abbott directional DBS 4-contact directional electrodes 6172 Infinity) because of medication-resistant severe flexion–extension head and trunk tremor, associated with mild signs of cervical dystonia (retrocollis, left laterocollis) since the age of 54. She also presented with ataxic gait from the age of 56. No signs of postural or intention tremor were present in the upper and distal limbs. There was no family history of movement disorders. Brain MRI was unremarkable and neuropsychological testing was normal. Imaging of the dopamine transporter was negative. Genetic tests ruled out spinocerebellar ataxias (types 1, 2, 3, 6, 12, 14, 17, and 35), Friedrich’s ataxia. Next-generation sequencing for gene variants associated with isolated and combined dystonia was negative (see list of genes examined in supplementary material, Online Resource 1).

In this patient, we opted to target the probabilistic coordinates of the ZI area under the anterior part of the thalamus, with a trajectory crossing the VO complex.

At visual examination, MER documented an irregular pattern with a mean discharge rate between 8–10 Hz, responsive to voluntary movement, bilaterally from 3.5 to 1.0 mm above the target. No cutaneous sensitive cells were recorded, nor were passive joints movement. From the target to 2 mm below, electrical silence and only sparse cells were recorded. IOM revealed no adverse capsular effects or paresthesias. The final tip of the macroelectrode was positioned 1.7 mm below the calculated target (Table 1). No surgical adverse events were recorded on cerebral MRI performed 12 h after DBS surgery (images available in supplementary material, Online Resource 1). Early and significant alleviation of tremor was documented 12 h post-electrode implantation, before IPG positioning, as a possible microlesional effect (see video in supplementary material, Online Resource 2).

During monopolar revision of each contact, the best head tremor control was achieved at 4.0 mA amplitude and 130 Hz frequency with the two intermediate segmented contacts (Table 2). No stimulus-related side effects were observed. Based on MER and IOM, we decided to stimulate the ventral segmented contact on the left hemisphere and the rostral segmented contact on the right hemisphere, both in ring mode fashion. After a 1-month trial at 130 Hz frequency, 60 μs pulse width and up to 4.0 mA amplitude, the patient showed suboptimal tremor control and experienced dysarthria at 3.3 mA. Consequently, we empirically switched to a 185 Hz frequency and lowered the amplitude to 2.8 mA using directional contacts to achieve a wider therapeutic window. Final stimulation parameters are reported in Table 2. These settings provided significant tremor reduction, improvement of soft dystonic signs, and no worsening of ataxia up at 6-month follow-up (Fahn–Tolosa–Marin Rating Scale, FTMRS:baseline = 44/144; 6 months = 14/144; Burke–Fahn–Marsden Rating Scale movement scale, BFMRS-M: baseline = 8/120;6 months = 4/120) (video in supplementary material, Online Resource 2). Control of tremor and dystonia was maintained at 6-year follow-up.

**Table 2:** Stimulation parameters at different times of follow-up

	Time from surgery	Electrode (side)	Contacts (% of current)	Amplitude (mA)	Pulse width (µs)	Frequency (Hz)
Case 1	6-month follow-up	Right	5 (33%) 6 (33%) 7 (34%)	3.5	60	185
		Left	2 (33%) 3 (33%) 4 (33%)	3.2	60	185
	6-year follow-up	Right	3 (25%) 5 (25%) 6 (25%) 7 (25%)	2.8	60	185
		Left	2 (33%) 3 (33%) 4 (33%)	2.8	60	185
Case 2	6-month follow-up	Right	1 (42%) 8 (58%)	4.3	60	185
		Left	1 (30%) 8 (70%)	4.9	60	185
	3-year follow-up	Right	1 (42%) 8 (58%)	4.9	60	185
		Left	1 (30%) 8 (70%)	5.2	60	185
Case 3	6-month follow-up	Right	3 (32%) 6 (68%)	2.6	60	185
		Left	1 (15%) 2 (10%) 4 (52%) 7 (23%)	3.6	60	185

For case 1, Abbott lead numeration was transformed as follows: 10b = 3, 11a = 5, 11b = 6, 11c = 7, 2a = 2, 2b = 3, 2c = 4

In the 3D rendering, left and right leads crossed the entire VOa, while the VTA appeared to spread to the ZI, particularly on the left side (Fig. 2).

### Case 2

The 47-year-old man underwent bilateral DBS implantation targeting VOa/VOp and ZI (Boston directional DBS 4-contact directional electrodes) due to medication-resistant head (yes-yes pattern) and truncal tremor with prominent involvement of the abdominal region. This was associated with truncal dystonia and mild cervical dystonia (right laterocollis), which began at age 44. He had experienced mild left lower limb dystonic posture since age 15, initially presenting as foot inversion during gait and later at rest, with the occurrence of tremor in the right hand 3 years later. Brain MRI and neuropsychological testing were unremarkable. Family history was non-informative. Genetic testing revealed a positive TOR1A (DYT1) heterozygous mutation.

In this case, we opted to target the probabilistic coordinates of VOa–VOp complex and steered the trajectory to ZI region, caudally and medially to the VO complex (Table 1).

MER recorded bilaterally irregular patterns in bursting mode from 6.0 mm to 1.5 mm above the target, with a mean frequency of 15–45 Hz. Electrical silence was documented from 1.5 mm above to 1.0 mm below the target, with a small zone of 0.5 mm length where cells discharged in a regular pattern at high amplitude at 20–40 Hz. No passive joint movement, voluntary movement nor cutaneous sensitive cells were documented. IOM revealed no side effects and tremor control with intraoperative microstimulation was achieved with the final anterior track bilaterally at 5.0 mA. This outcome was confirmed with macrostimulation at 4.0 mA. The final electrode was positioned 2 mm below the calculated target (Table 1). No surgical adverse events were documented.

The monopolar review revealed no adverse effects up to 5.5 mA at the first and last contacts, with the best tremor control at the caudal contact. We decided to stimulate at a fixed pulse width of 60 μs and a fixed frequency of 130 Hz, using both the caudal and rostral contacts in ring mode, incrementing the voltage until tremor control. Increasing amplitude to 5.5 mA was insufficient to completely control truncal tremor, and the patient reported mild facial muscle contractions. Based on the previous experience of the first case, we switched to a 185 Hz frequency using the same contacts, while lowering the amplitude to 4.3 mA on the right electrode and 4.9 mA on the left. This adjustment determined nearly total tremor control without side effects (Table 2 and video in supplementary material, Online Resource 3). With these stimulation parameters**,** the patient experienced a significant and early reduction in segmental tremor (FTMRS at baseline 58/144, at 6 months and 3 years 3/144) and good improvement of laterocollis and left lower limb dystonic posture (BFMRS-M at baseline 20/120, at 3 years 4/120), maintained after 3 years of stimulation (see video in supplementary material, Online Resource 3**).**

In the 3D rendering, left and right leads crossed minimally through VOa and VTA mostly involved r-ZI. No adverse events or side effects were reported in short- and long-term follow-up (Fig. 2).

### Case 3

This 44-year-old man underwent bilateral DBS targeting VOa/VOp and ZI (Boston Gevia directional DBS 4-contact directional electrodes) due to due to cervical dystonia manifesting with prominent head tremor and poorly responsive to medications and botulinum toxin**.** He had cervical dystonia since age 20, with subsequent progressive involvement of the right body side and trunk and occurrence of tremor in the right upper limb 10 years later. Brain MRI, imaging of the dopamine transporter, and cognitive function tests were normal. There were no neuropsychiatric symptoms, and family history was non-informative. Next-generation sequencing for gene variants associated with isolated and combined dystonia was negative (complete list of dystonic genes examined in supplementary material, Online Resource 1).

In this case, we opted to target the probabilistic coordinates of VOa–VOp complex and steered the trajectory to ZI region, caudally and medially to the VO complex (Table 1).

In the posterior tracks, MER bilaterally showed a regular pattern (mean frequency 18–22 Hz) responsive to kinesthetic/passive joint movement from 4.5 mm above the target, occasionally interrupted by typical tremor cells. The central and anterior tracks showed sporadic cells discharging in burst mode (5–9 Hz) not responsive to cutaneous stimulus and/or voluntary or passive movement. Beyond the target, all tracks exhibited electrical silence for a length of 2.5 mm. IOM bilaterally revealed capsular effects (facial muscle contractions) in the anterior tracks and paresthesias with the posterior ones. Considering the MER in posterior tracks compatible with VIM pattern and the presence of capsular effects with the anterior tracks, we decided to place the DBS leads in the central tracks bilaterally (Table 1). Intraoperative macrostimulation revealed complete tremor suppression in the right upper limb at 2.5 mA with the two directional contacts in ring mode activation.

After monopolar review of each contact (no side effects until 3.5 mA with the best tremor control using the two intermediate segmented contacts), we engaged these contacts in directional mode, starting with high-frequency stimulation at 185 Hz until tremor control at 3.6 mA. Subsequently, we steered stimulation to the caudal ring contact, achieving further improvement in tremor control and dystonic posture without side effects (see video in supplementary material, Online Resource 4).

After the first month of stimulation of VOa/VOp and ZI, the patient experienced a remarkable improvement in segmental tremor, confirmed at 6-month follow-up (FTMRS baseline = 29/144, FTMRS 6-month = 6/144) and dystonic postures (BFMRS-Mbaseline = 30/120, BFMRS-M 6-month = 4/120). No residual head tremor was detectable and no stimulus-related side effects occurred (video in supplementary material, Online Resource 4).

In the 3D rendering, leads bilaterally crossed the VO complex and the VTA involved VOa, VOp and minimally also r-ZI (Fig. 2).

## Discussion

This is the first case series reporting clinical outcomes and benefits in three forms of Midline Tremor treated with DBS, targeting the VO complex and ZI. In our three cases, no adverse events secondary to surgery and/or stimulation were observed, nor were tolerance effects [9, 31].

In the first case, we achieved good control of head tremor within the first months, with benefits remaining stable at the 6-year follow-up. This subject had a combined tremor syndrome characterized by prominent Midline Tremor associated to gait ataxia and soft dystonic signs [32, 33]. Considering this mixed phenotype and the presence of gait ataxia, we avoided VIM-DBS, supported by literature reports [34, 35]. We targeted the more anterior and medial VO complex, avoiding direct stimulation of the ascending cerebello-thalamic tract (CTT) in the caudal ZI, likely preventing worsening of gait ataxia, as reported as adverse event after VIM/c-ZI DBS [36]. Predominant VOa stimulation, involving pallido-thalamic pathway, resulted in good and sustained control of action tremor and cervical dystonia.

In the second case, with a 3-year follow-up, the genetic form of TOR1A suggested the GPi as a target due to the well-documented significant long-term outcomes in these genetic forms of isolated dystonia. However, we focused on the most disabling symptom, midline DT, and chose the VO complex with the opportunity to involve also ZI if needed. This case resulted in early and sustained improvement in both dystonia and DT, with preferential stimulation of ZI.

In the third case, the choice of combined targeting was dictated by the predominant disabling symptoms, cervical and trunk dystonia associated with dystonic head and arm tremor. Intraoperative testing was crucial in accurately delineating the VOa–VOp complex and avoiding VIM and internal capsule. The patient experienced immediate post-surgical control of DT and cervical-trunk dystonia, which remained stable at the 6-month follow-up.

The precise mechanisms by which this novel targeting and stimulation paradigm positively affect DT are still unknown, given the intricate anatomy and pathophysiology of this subcortical network. The Ventral-Oralis (VO) complex, composed of the Ventral Oralis anterior (VOa) and Ventral Oralis posterior (VOp), lies immediately anterior and slightly medial to the VIM nucleus of the thalamus. Ventral to VOp is the prelemniscal radiation (RAPRL), a white matter region that contains fibers arriving from the cerebellum (the dentato-rubro-thalamic tract, DRTT) directed to VIM. Latero-anteriorly to RAPRL and beneath the thalamus is ZI, an elongated structure extending rostrally above and medial to the STN, and caudally behind the STN. These anatomical structures are both difficult to identify with standard MRI and even atlas-based indirect targeting shows significant variations due to individual anatomical differences. Considering the bicommissural plane, VO complex is selected from 10 to 13.5 mm lateral to the AC-PC line, from 2 mm anterior to 3 mm posterior to the midcommissural point, and from 0.5 mm below to 2 mm above the bicommissural plane. Even more variability is reported for ZI area, often refined using the Red Nucleus (RN) and STN landmarks. In awake conditions, side effects of VO complex and ZI intraoperative stimulation are infrequent, including speech difficulties, transient paresthesias, muscle spasms, dizziness, or blurred vision, mostly when the target is too medial or anterior [17, 21, 37].

In our series, target selection and trajectory were based on the bicommissural plane, anatomical landmarks detected on stereotactic MRI, and refined by the intraoperative recording and the absence of intraoperative side effects.

The recent interest in the thalamic Ventral-Oralis complex (VO complex) as a target for DBS for tremor syndromes can be attributed to its key role in the cortex-basal ganglia-thalamus-cortex loop related to motor functions [12, 37, 38]. Notably, the pallido-thalamic (via the fasciculus thalamicus) and cerebello-thalamic afferents are significantly intertwined within the VO complex, with VOa predominantly associated with the pallido-thalamic pathway and VOp with the cerebello-thalamic pathway. This peculiar interconnection explains why the VO complex should be considered a viable alternative to GPi and/or VIM in DT.

ZI-DBS is an emerging treatment [13, 16–23] for various movement disorders syndromes. Its proximity to thalamic and subthalamic nuclei and pallido and cerebello-thalamic passing fibers makes it an ideal target in tremor syndromes of different etiologies [23, 37]. Regarding ZI, anatomical targeting is first necessary to distinguish between the rostral and caudal areas. DBS of the c-ZI region has shown efficacy in various tremor syndromes, especially in those ET with a prominent proximal limb involvement, as well as Parkinson’s disease (PD) and possibly in cervical dystonia with DT [19, 20]. Some case series [36, 39, 40] on different tremor syndromes (mostly secondary: post-ischemic or post-traumatic) reported double targeting with the same electrode stimulating VIM or VOa/VOp and c-ZI. Sitsapesan et al. reported eight cases of post-traumatic tremor, four of them treated with stimulation of VOp and ZI [21], achieving an overall tremor reduction of 80.75%, but noting an exacerbation of pre-existing gait ataxia in one patient and transient paresthesias in another. However, details about electrode characteristics and alignment of these two distant targets were not clearly described (c-ZI is ~ 3 mm below the VOa/VOp, and the distance between VO complex and c-ZI is ~ 12 mm) [20, 36]. Current commercially available electrodes (4-contacts electrodes each of 1.5 mm length with an interspace length of 0,5 mm) make it challenging to reach both VO complex and c-ZI with the same electrode. More likely, the last contact could involve the r-ZI, which is under the ventral border of thalamus with an estimated diameter of ~ 1 mm [20]. Only with an 8-contact electrode could it be possible to properly align thalamic nuclei and PSA, as reported by dos Santos Gilardi’s case report [36].

Data on r-ZI stimulation are scarce, and existing reports describe some beneficial effects in parkinsonian tremor and levodopa-induced dyskinesias/dystonias [13, 41, 42]. There are no reports on the possible effects of r-ZI stimulation on non-parkinsonian tremor or dystonia. For dystonia, it is conceivable that r-ZI-DBS exerts its efficacy through involvement of pallido-thalamic fibers, although how it modulates DT is less clear. In DT, it is conceivable that tremor control is obtained through the involvement of the same pallido-thalamic fibers which surround the r-ZI (the lenticular fasciculus and the ansa lenticularis are fused to form the thalamic fasciculus) and run medial and superior to it. Indeed, as recently shown by Tsuboi et al. [4], in 20 patients with DT, the pallido-thalamic tract projects primarily to the VOp, which in turn modulates the cerebello-thalamic fibers. The involvement of both dysfunctional circuits might explain the good response in DT**.** An alternative hypothesis, as suggested in the pathophysiology of resting tremor in PD [43], is that the involvement of pallido-thalamic fibers might trigger tremor episodes even in non-parkinsonian tremor syndromes. Alternatively, it is conceivable that r-ZI region stimulation might directly modulate the subthalamic portion of the DRTT when its widespread fibers pass through the anatomical “bottleneck” of the ZI [12, 23, 44, 45].

Furthermore, ZI and/or pallido-thalamic fibers could exert a crucial role in controlling the Midline Tremor since, as increasing evidence shows, ZI is responsible for the generation of axial and proximal limb movements [46–49], while the predominant control of distal muscles is exerted by the thalamo-cortical pathways [35]. Recent cases of DBS of the pallidal network for Midline Tremor seem to support this hypothesis [7, 50].

In this study, we observed early and sustained control of both dystonia and tremor in all patients. Rapidity of onset of DBS benefit has been described for subthalamic nucleus stimulation in dystonia and might be also a feature of thalamic targets. Large cohort studies are needed to confirm this finding and postulate a mechanism.

In this novel DBS-programming approach, given the absence of previously coded algorithms, we adopted an empirical “shotgun” method. This involved testing each electrode configuration and stimulation parameters until optimal symptom control was achieved. Although considered outdated due to its time-consuming nature, this approach allows for the testing of novel paradigms compared to traditional VIM or GPi programming. The high frequency and voltage required to achieve tremor control without side effects, previously suggested only in the seminal works of Coubes et al. [26] for dystonic patients, are quite unique, given the small volume of these two structures. High-frequency stimulation likely allowed for a decrease in the range of voltage needed to evoke tremor suppression without causing capsular side effects [51, 52]. However, the pathway recruitment dynamics associated with VO complex and ZI DBS remain speculative, as reported in a recent computational line of research [23].

We acknowledge the limits of this pilot, open-label study, primarily the small sample size. The rare nature of the tremor syndrome and the uncommon target chosen made it challenging to select a more conspicuous sample size in a single-center study. Moreover, we know that the choice of the target is still not fully standardized. MRI direct identification is still not completely reliable, and our choice of target coordinates is derived by combining indirect and direct targets. On the other hand, intraoperative MER and IOM added significant information by characterizing the physiology of the neuronal environment and helped to refine better the final electrode position. Indeed, in all cases, the final electrode position and active contact chosen, based on intraoperative testing, was confirmed by the subsequent feedback from VTA calculation, the optimal clinical outcome, and the absence of stimulus-induced side effects.

Despite these limitations, we think that such significant preliminary results reported in our small case series are interesting, but certainly this data should be confirmed in a larger sample of patients, possibly in a multicenter study.

## Conclusion

In the syndromes of Midline Tremor variably associated with dystonia, identifying the best target for DBS might be challenging. Instead of assuming two different consecutive interventions in VIM and GPi or vice versa, with a final implantation of four electrodes as suggested elsewhere [8, 10, 53], we believe, as reported in other case reports [34, 54], that VO complex/ZI may be a viable and safe option even in specific forms of tremor with axial distribution.

## Supplementary Information

Below is the link to the electronic supplementary material.

Supplementary file1 (DOCX 1448 KB)

Supplementary file2 (MP4 301149 KB)

Supplementary file3 (MP4 399115 KB)

Supplementary file4 (MP4 406118 KB)

## Data Availability

The authors confirm that the data supporting the findings of this study are available within the article and its supplementary materials.
