# Supplementary material for: Thalamic ventral-Oralis complex/rostral zona incerta deep brain stimulation for midline tremor
Source: J Neurol. 2024 Aug 10;271(10):6628–38. doi: 10.1007/s00415-024-12619-3 (PMC11447151; doi:10.1007/s00415-024-12619-3)
Supplement: Supplementary file 1 — Supplementary file1 (DOCX 1448 KB) [file 415_2024_12619_MOESM1_ESM.docx]

Title: **THALAMIC VENTRAL ORALIS COMPLEX/ROSTRAL ZONA INCERTA DEEP BRAIN STIMULATION FOR MIDLINE TREMOR.**

Journal**: Journal of Neurology**

**Authors**: Alba Scerrati MD1,2, Andrea Gozzi *MD3, Michele Alessandro Cavallo MD1,2, Giorgio Mantovani MD1,2, Pietro Antenucci MD3 , Chiara Angelini MD1,2, Jay Guido Capone MD3, Pasquale De Bonis MD, PhD1,2, Francesca Morgante MD,PhD4, Vittorio Rispoli MD5, Mariachiara Sensi MD3

**Affiliations**:

1 Department of Translational Medicine, University of Ferrara, Ferrara, Italy

2 Neurosurgery Department, S. Anna University Hospital of Ferrara, Ferrara, Italy

3 Neurology Department, S. Anna University Hospital of Ferrara, Ferrara, Italy

4 Neurosciences and Cell Biology Institute, Neuromodulation and Motor Control

Section, St George’s University of London, London, UK

5 Neurology, Neuroscience Head Neck Department, University of Modena and

Reggio Emilia, Modena, Italy

***Corresponding Author’s** name and current institution: Andrea Gozzi MD (ORCID: 000000025248791X)

Neurology Department, S. Anna University Hospital of Ferrara,

Ferrara, Italy

Via Aldo Moro 8, 44124,

Corresponding Author’s Email: [andrea01.gozzi@edu.unife.it](mailto:andrea01.gozzi@edu.unife.it)

**Supplementary material**

**Complete list of dystonic genes examined in all patients:** ADCYS, AN03, ATM, ATP1A3, ATP78, CACNA1A, CIZ1, GCH1, GLB1, GNAL, GNA01, GRIN1, HPCA, KCNMA1**,** NKX2-1, fDE10A, PDE2A, PNKD, PNKP, PRKRA, PRRT2,, SCNBA, SCP2, SETX, SCGE, SLC2A1, SLC39A8, SYT1, TH, THAP1, TIMMBA, TOR1A, TUBB4A, VPS16, COL6A3, DHDDS, KCTD17, KMT2B, RIMS1, RIMS2, TAF1, TBC1D24.

**Supplementary Figure** Axial sequential T2-weighted MRI brain images in the cranio-caudal direction. From top to bottom: case 1, case 2, case 3

**
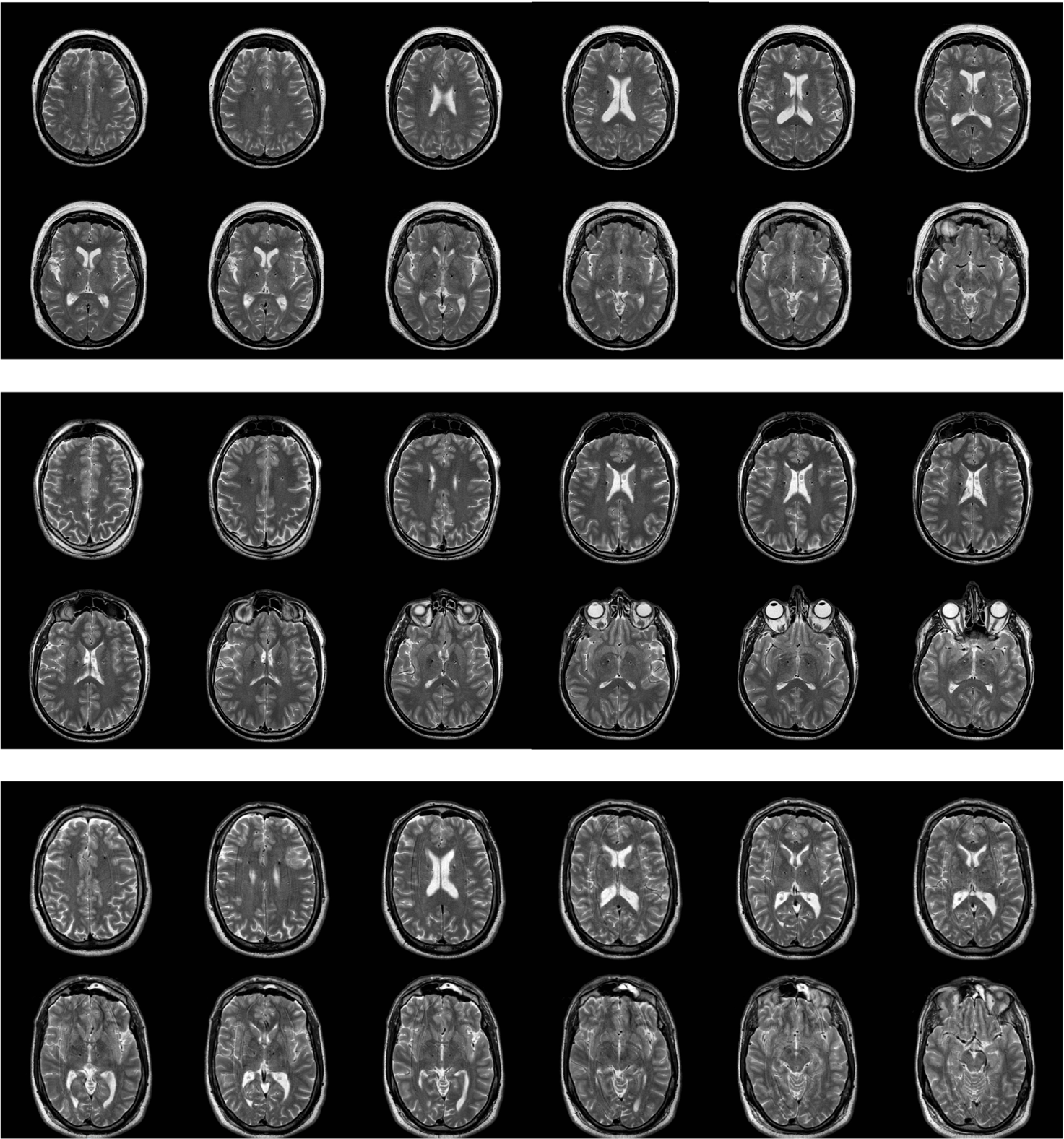
**
